# Emergency workers’ experiences of the use of section 136 of the Mental Health Act 1983: interpretative phenomenological investigation

**DOI:** 10.1192/bjb.2020.21

**Published:** 2020-12

**Authors:** Mirella Genziani, Steve Gillard, Lana Samuels, Mary Chambers

**Affiliations:** 1 Faculty of Health, Social Care and Education, Kingston University and St George's, University of London, UK; 2 Population Health Research Institute, St George's, University of London, UK; 3 Independent Mental Health Researcher, UK

**Keywords:** Mental Health Act 1983, section 136, interpretative phenomenological analysis, decision-making, pre-hospital care

## Abstract

**Aims and method:**

To explore the experiences of emergency workers dealing with incidents in which section 136 of the Mental Health Act 1983 is invoked by the police. Data from interviews with police officers and ambulance workers in a London locality were subject to interpretative phenomenological analysis.

**Results:**

Participants felt they were the first port of call and that training should be improved to help them deal with those experiencing mental health crises in the community. Police participants noted time pressures trying to gain individuals’ trust and described section 136 detention as sometimes feeling like a betrayal of the individual. Most participants had negative experiences of admissions to the 136 suite; several suggested ways of improving the admissions system. Several went beyond their expected duties to ensure that distressed individuals were supported before accessing mental healthcare services.

**Clinical implications:**

Improving training of emergency workers in dealing with mental health crises would also help with aftercare decision-making. Learning identified from the participants’ experiences lends support to collaboration between emergency and mental health services, an important step towards improving the section 136 process so that detainees can access help without unnecessary delay.

Section 136 of the Mental Health Act 1983 for England and Wales (amended in 2007) permits police officers to legally detain someone who is in a public place and is believed to be at risk to themselves or others and to be requiring immediate care or control.^1^ Detainees are taken by police and the ambulance service to a designated place of safety for assessment, with arrangements for treatment and aftercare.^1,2^ According to the Act, a ‘place of safety’ could be a police station, a unit in a psychiatric hospital or another suitable residential place willing to temporarily accommodate a detained person.^2,3^

Implementing section 136 not only requires quick thinking but can pose a challenge for emergency workers with limited or no training in mental health.^1,4,5^

Two papers^6,7^ exploring police officers’ experiences of section 136 highlighted participants’ concerns about vulnerable individuals being turned away from in-patient mental health services. There is a paucity of research on the experiences of ambulance and paramedic workers. This study aims to fill this gap by exploring emergency workers’ experiences of section 136 and how they make sense of them.

## Method

### Participants

Emergency workers were purposively sampled on the basis of the roles they undertake and their experiences of implementing section 136. Four individuals from a police force and three from an ambulance complex in a London locality participated. This sample size is consistent with the interpretative phenomenological analysis (IPA) literature, which suggests the appropriateness of relatively small samples, for whom the research questions are significant.^8,9,10^

Characteristics of the participants (who were allocated anonymised identifiers such as PO1) are presented in Table 1.

**Table 1:** Participant characteristics at time of interview

Participant identifier	Role	Gender
Police 1 (PO1)	Police officer	Male
Police 2 (PO2)	Police officer	Female
Police 3 (PO3)	Police officer	Male
Police 4 (PO4)	Police mental health liaison officer	Male
Ambulance worker 1 (AMB1)	Ambulance worker	Female
Ambulance worker 2 (AMB2)	Ambulance worker	Male
Ambulance worker 3 (AMB3)	Paramedic	Female

### Ethics

This research received ethical approval from the London South East National Health Service Research Ethics Committee in 2011, reference number 11/LO/1002. Participants were given a detailed information sheet and completed a written consent form.

### Data collection and analysis

Police officers (*n* = 4) and ambulance workers (*n* = 3), not known to each other, participated in separate interviews (data were collected between 2012 and 2014). In keeping with the IPA approach, open-ended questions were used inviting participants to tell their stories in their own words, with prompts where necessary.^8,11^

The following are examples of questions asked.

**Ambulance workers**: ‘Please could you tell us about your recent experiences of taking someone under a section 136 from a public place to a place of safety?’. Prompt: ‘How do you feel about dealing with incidents of this nature?’.

**Police officers**: ‘Please could you tell us about a recent experience of sectioning someone?’. Prompts: ‘What factors are involved in making this decision? Did you feel that your action(s) were justified?’; ‘Have your encounters with individuals in these kinds of situations affected, if at all, the way you view people with mental illness?’.

Interviews took place at police stations or ambulance complexes and lasted around 50 min. The third author (L.S.) took summary notes of the interviews to supplement the audio transcripts. Responses were digitally audio-recorded and transcribed into text verbatim by the first author (M.G.).

A framework underpinned by the IPA literature was developed and used to inform the analysis process.^5,8,10^ Questions were asked of the data by M.G. to aid the process of identifying themes: (a) What experiences are being shared and how are respondents making these meaningful to one another?; and (b) What are the consensus, conflicts/contradictions and how these are being managed?^12,13^

Themes were deemed to be prominent if they occurred in approximately one-third to half of participants’ accounts.^9,11^ For example, most participants referred to their experiences of interacting with individuals in distress, how they felt about engaging and showing empathy. This was deemed to be a shared experience and characterised by the theme: ‘Making yourself human as first responders’.^14,15^

Quality checks were undertaken by M.G. and L.S., which involved comparing a random selection of themes alongside verbatim quotes. This was done to ensure that themes closely reflected the participants’ experiences and it fits with the epistemological approach.^9,16^

The findings are presented under the thematic headings below. Quotes from the participants are interwoven with interpretative commentary that encompasses emotions evoked as well as the language and text they relate to.

## Results

### Making yourself human as first responders

Participants described themselves as the ‘*first* responders’ and spoke of how they approached and communicated with the individuals involved. Some were sensitive to the way a uniform or emergency vehicle can be perceived. Communication was seen by these participants as ‘Essential for people who may be suffering from some sort of psychosis, sort of gaining trust, turn your radios down, taking off your hat. Essentially try and be the only person talking to them’ (PO3).

Police participants reflected that detaining and removing someone against their will could generate feelings of betrayal towards the person they were trying to build trust with. The main challenge for them was in encouraging individuals to accept help voluntarily. Some of the police officers felt that they did their best to demonstrate that their role is not only about enforcing the law, but also supporting individuals in difficult and sensitive circumstances:

‘It takes a good few hours to build rapport with her, which can be difficult if someone is going through an episode. You don't want them to feel humiliated or mocked. Because you want them to know you are there to help’ (PO1).

Participants tried to make sense of the barriers and pressures that made it difficult for them to establish a rapport:

‘Making yourself more sort of human to them. But the problem is your lifeline is your radio and so by turning it down you are not hearing what's happening outside’ (PO2).

‘We are under pressure from our radio, from our supervisors. So, it sounds awful but it is time-consuming. In this day and age we do not have 2 hours spare, which sounds awful to say. But you end up having to build that rapport up, say “come on you know what, the best thing to help you is to voluntarily go with the ambulance”. And probably 7 out of 10 it works’ (PO2).

The excerpt below is a good example of the police officer involving a family member to support a distressed individual. It brought about a sense of reassurance for the parties involved in ensuring that the person was supported in keeping themselves safe:

‘Like, the lady, she was holding a knife to her throat. We ended up calling her son to say “look, your mum, she's voluntarily said she can come to hospital. This is what's happened. We just want you to be aware because I think she could do with a loving face, like have someone from the family there”’ (PO3).

### The 136 suite

The place of safety or ‘136 suite’ was salient for the participants as the transition point into in-patient mental healthcare. All of the participants spoke less favourably of their experiences of trying to get individuals admitted into the suite. Strong feelings were voiced regarding the potential welfare of detainees in situations where there were delays. Ambulance workers felt for individuals who had little or no choice but to wait inside an emergency vehicle: ‘I find once you get to [hospital X], I've waited 2 hours before to get into the 136 suite and that can cause issues with the patient’ (AMB2).

When considering what the waiting might involve, the ambulance workers reflected that ‘We're not allowed in the building. So, we just literally sit outside in the ambulance or in the police van’ (AMB2).

Unlike ambulance workers, police officers were able to enter the 136 suite and liaise with staff there. Ambulance worker participants often speculated on what goes on inside the facility, which may have generated some friction between the ambulance workers and mental health workers at the 136 suite. They wondered what happened to individuals who were admitted and how they got on: ‘Once we take someone to [hospital X], we don't see what happens’ (AMB2).

The exchange below illustrates the police liaising with mental health workers at a 136 suite. There is a sense of the parties colluding with each other, together with somewhat blurred boundaries around responsibility. The liaison between police and hospital staff amplifies a sense of being in an awkward position and of feeling overwhelmed, in a situation in which there are no clear answers regarding care decisions:

‘It went back and forth, our inspectors got involved. Hospital staff basically turned around to us and said that if we left the hospital they would let him walk out of the hospital and into the street, where he would then become our responsibility again […] And that if he was to attack someone it would be on the basis that the police left him and that they washed their hands of him […] We explained that we had no powers, essentially the only thing we had was a breach of the peace, inside the facility whilst he was there […] This went on for about 2 hours, this debate’ (PO1).

Most of the participants' experiences at the point of entry to the 136 suite were negative. However, there was a unique example in which an individual under a section 136 was able to access the care they needed, in a timely and seamless manner:

‘I think I had a positive one. We had a gentleman who was out on the street. The police were already there when we arrived on the scene. And it was called in by friends of his because he was behaving erratically […] When we got there he was in the back of a police van, but his behaviour was quite self-harming, even though he was quite chatty […] And then what we've got in place was that a paramedic travelled with the police in the van to ensure the safety and the care for the patient. And actually we did take him straight to the 136 suite on this incident and he was booked straight in. So that worked really well. But that was during the day. It was early. It was a weekday. So everything was in place on that incident’ (AMB1).

### Training and quest for collaboration

When participants reflected on their skills in dealing with individuals experiencing mental health problems, there was a unanimous view that training and opportunities were somewhat limited. The participants reflected on specific areas that they felt could influence their practice, given their involvement in section 136 detentions. For example, ambulance workers felt that they would benefit from a better understanding of substance misuse and mental illness:

‘I'd like to have more training […] Historically, our training has been a bit ad hoc […] I think it's got to be around drugs and alcohol, what that impact is on how we are assessing a patient. Because as you can imagine, probably 75 per cent will have drugs and alcohol on board’ (AMB2).

‘There is no training on what you might want to look for, how it might present itself and different types of mental health, erm, issues. I don't think there were any role-plays or anything like that, which I think could possibly be helpful’ (PO3).

Ambulance workers put forth some practical suggestions that they felt would benefit professionals and detainees, with an emerging consensus towards a more collaborative approach. One respondent referred to the potential of a bed management system that he had found successful in general accident and emergency (A&E) settings:

‘A bed manager in A&E is always well versed on what beds are available. So ITU beds, neonate beds […] Why isn't that available in psychiatric healthcare?’ (AMB2).

The same respondent speculated as to whether such a system could be applied in the 136 suite to ease the transition into hospital care:

‘So, we're on a job with someone who is going to get 136’d […] So rather than waste half an hour with the police trying to ring the 136 suite, because they are obviously busy preparing for two to come in, why don't we get someone to just say “There's no beds. Your nearest bed is there”. Bang! Why can't we do that?’ (AMB2).

Further suggestions were put forth to minimise waiting times and ensure that detainees were promptly received and attended to at the 136 suite:

‘It needs some immediate action. It's not something that can be delayed […] Can we go early with the information that we're going to be taking a patient there?’ (AMB3).

In some cases, there was a sense of commitment and willingness to go beyond the remit of their roles, for example:

‘If we're going to be spending this long with patients […] waiting to convey them to the 136 suite, if we can find out more information on the scene then let's do it’ (AMB2).

## Discussion

This research is the first of its kind to collectively explore and combine findings from police and ambulance workers’ experiences of detaining individuals under section 136 of the Mental Health Act (England and Wales).^1^ This piece of work highlights that lived experience plays a key role in service development in a range of settings. Two key aspects of experience emerged from the findings: (a) therapeutic engagement in a crisis and (b) drawing on the expertise and experiences of the parties involved in a section 136 admission.

### Personal engagement

Professionals felt that how they approached and engaged with individuals had made some difference in those people's willingness to accept help. Police participants felt pressured by their agency to prioritise other emergencies over mental health incidents.^17,18^ It is not entirely clear how much time emergency workers can devote to situations in which mental health problems are suspected. This was a source of conflict for study participants. Therapeutically engaging and gaining trust were seen by participants as an important first step and in the detainee's best interests as a way of enabling them to access immediate support.

### Accessing the 136 suite

A pressing concern for participants was the inordinate amount of delay regarding decisions on granting access to the 136 suite (the place of safety). In some cases, detainees were having to wait for longer than necessary inside an emergency vehicle or were refused entry, which generated further distress. The findings also point to friction between the emergency and mental health services regarding responsibilities of care. The collusion between the parties can have implications for the waiting time for detainees in need of immediate care and support. These findings were similarly noted in Burgess *et al*^5^ and Riley *et al*^6^ and is at odds with key recommendations from the Royal College of Psychiatrists’ section 136 national guidance. According to these guidelines: 136 suites should agree to accept an individual before the emergency services begin their journey and have the necessary staff on hand to receive individuals without delay or recourse to emergency professionals.^2,17^ In contrast, another finding conveyed an emergency worker's experience where the section 136 journey for the detainee in question was smoother and well supported. This finding highlights what one can learn from personal experiences and consider how these can inform future practice. A prominent aspect to this study was that emergency workers wanted to be more involved in the section 136 process and to work jointly with staff at the 136 suite. This was evidenced by their efforts to seek out practical solutions for the dilemmas they experienced. The participants felt that this way of working would enhance the quality of the experience for detainees accessing mental healthcare.^10,19^

### Training

This study has shown that ambulance and paramedic workers play a pivotal role in dealing with individuals experiencing mental health problems in a community setting. Yet, training for this group of professionals is somewhat limited. There was a general consensus regarding the improvement of training to enable emergency workers to feel more confident in recognising how mental health problems can present, dealing with crises and engaging with individuals affected.^6,14,19^

Given this, future work needs to prioritise interdisciplinary training to enable the various agencies to appreciate the roles and limitations of their services. These different agencies can learn a lot from each other.^20^ In keeping with suggestions in previous work, involvement of patients and carers could enhance the quality of the training for emergency workers, by bringing in their lived experiences.^7,20^ This is another important area, which would benefit from being further explored in future research.

### Strengths and limitations

Interpretative phenomenological analysis (IPA) captured emergency workers’ experiences of the section 136 process and how they were affected by it. Situating the study in a catchment area of a National Health Service mental health trust in London could be seen as both a strength and a weakness, since the views expressed only reflect those who took part in the study. It is possible that the views of emergency workers with different characteristics in other areas of England and Wales will vary. Further research in other geographical areas could help to ascertain whether this perspective of section 136 detainment could be understood more widely.

## Data Availability

Data are available from the authors.

## About the authors

**Mirella Genziani**, BSc, MSc, PhD, PGDip CBT, is a Cognitive Behavioural Psychotherapist in the Faculty of Health, Social Care and Education at Kingston University and St George's, University of London, UK. **Steve Gillard**, BSc, PhD, is a Reader in Social and Community Mental Health in the Population Health Research Institute at St George's, University of London, UK. **Lana Samuels**, is an Independent Mental Health Researcher. **Mary Chambers**, RN, BEd, PhD, is Professor of Mental Health Nursing in the Faculty of Health, Social Care and Education, and Director of the Centre for Public Engagement, at Kingston University and St George's, University of London, UK.
