# Emergency workers' experiences of the use of section 136 of the Mental Health Act 1983: interpretative phenomenological investigation – ERRATUM

**DOI:** 10.1192/bjb.2020.141

**Published:** 2022-08

**Authors:** Mirella Genziani, Steve Gillard, Lana Samuels, Mary Chambers

In the original published article, the country name was missing from a co-author's affiliation. This has now been corrected.
